# Urban–rural disparity in utilization of preventive care services in China

**DOI:** 10.1097/MD.0000000000004783

**Published:** 2016-09-16

**Authors:** Xiang Liu, Ningxiu Li, Chaojie Liu, Xiaohui Ren, Danping Liu, Bo Gao, Yuanyuan Liu

**Affiliations:** a Department of Health and Social Behavior, School of Public Health, Sichuan University, Chengdu, Sichuan Province, China; b School of Public Health, La Trobe University, Melbourne, Victoria, Australia; c Department of Epidemiology and Health Statistics, School of Public Health, Sichuan University, Chengdu, Sichuan Province, China.

**Keywords:** preventive care services, urban–rural disparity, utilization of health care

## Abstract

Preventive care service is considered pivotal on the background of demographic ageing and a rise in chronic diseases in China. The disparity in utilization of preventive care services between urban and rural in China is a serious issue. In this paper, we explored factors associated with urban–rural disparity in utilization of preventive care services in China, and determined how much of the urban–rural disparity was attributable to each determinant of utilization in preventive care services. Using representative sample data from China Health and Nutrition Survey in 2011 (N = 12,976), the present study performed multilevel logistic model to examine the factors that affected utilization of preventive care services in last 4 weeks. Blinder–Oaxaca decomposition method was applied to divide the utilization of preventive care disparity between urban and rural residents into a part that can be explained by differences in observed covariates and unobserved part. The percentage of rural residents utilizing preventive care service in last 4 weeks was lower than that of urban residents (5.1% vs 9.3%). Female, the aged, residents with higher education level and household income, residents reporting self-perceived illness in last 4 weeks and physician-diagnosed chronic disease had higher likelihood of utilizing preventive care services. Household income was the most important factor accounting for 26.6% of urban–rural disparities in utilization of preventive care services, followed by education (21.5%), self-perceived illness in last 4 weeks (7.8%), hypertension (4.4%), diabetes (3.3%), other chronic diseases (0.8%), and health insurance (−1.0%). Efforts to reduce financial barriers for low-income individuals who cannot afford preventive services, increasing awareness of the importance of obtaining preventive health services and providing more preventive health services covered by health insurance, may help to reduce the gap of preventive care services utilization between urban and rural.

## 1 Introduction

Preventive care helps find and stop health issues before people have any symptoms. On the background of ageing population and rise of chronic diseases in China, prevention is particularly more cost-effective than medical treatment.^[1]^ Preventive care services encompass a wide range of healthcare measures including routine check-ups, disease screenings, and immunizations, which can be undertaken to prevent the occurrence of disease and detect disease early.^[2]^ It has been documented that preventive care service utilization reduces premature mortality and improves quality of life.^[3,4]^ Underutilization of preventive care services may result in failures to identify treatable healthcare problems and prevent potentially life-threatening disease.

Utilization of preventive care services is affected by many factors, including individual factors (such as age, education, and income) and supply factors (such as allocation of resources and quality of service).^[5–9]^ Although China has achieved dramatic economic development during the last 3 decades, this economic development did not necessarily reduce inequality including individual factors and supply factors between urban and rural. Urban areas have traditionally had a significantly better healthcare system than rural areas. For example, according to China health statistics yearbook 2015, registered doctors available in urban communities as measured by per thousand populations were more than twice of those in rural communities: 2.57 versus 1.^[10]^ For rural populations, they have less education and lower income, are less likely to be insured, and have longer travel distance to their regular source of medical care when compared to urban residents.^[11,12]^ Due to the obvious gap between urban and rural in China, preventive care services may present more serious challenge for rural healthcare providers. However, the majority of researches about urban–rural disparity in healthcare have focused on access to and utilization of inpatient and outpatient medical care services,^[13–15]^ and only several studies explored the inequality in utilization of preventive care services. Chen et al^[16]^ and Yu^[17]^ have noted that rural residents were less likely to utilize preventive care services than urban residents in China, and these studies also showed that age, income, education, health insurance, and chronic disease were associated with utilization of preventive care. For example, family income has generally been recognized as a critical factor for utilization of health service. Urban–rural family income gap certainly led to urban–rural disparity in utilization of preventive care services. However no studies have further explored that how much or what percent of the urban–rural disparity in utilization of preventive care services was attributable to family income. Arguably, preventive care utilization may not completely be consistent with income due to its low-cost requirements. Studies in the developed countries have found that underutilization of preventive care is common even when it is free.^[18]^ Besides family income, other important factors contributing to the urban–rural disparity should be explored. Furthermore, we want to know which factor is relatively more important in accounting for the urban–rural disparity in utilization of preventive care services: differences in age, in educations, in income, in health insurance, etc.

Motivated by the above facts, we conducted this study using a Chinese national survey to examine urban–rural difference in utilization of preventive healthcare services, and to determine factors including sociodemographic and other characteristics associated with the difference. Furthermore, we applied Blinder–Oaxaca decomposition method to explore the contribution extent to which urban–rural disparity in utilization of preventive care services can be explained by differences in the observed indicators and unobserved component. Our study provided evidence to health policymakers for the reason of urban–rural disparity in utilization of preventive care services in China, and gave some advice to eliminate the unequal utilization.

## 2 Materials and methods

### 2.1 Study population

The present study used data from the China Health and Nutrition Survey in 2011 (CHNS 2011). CHNS is an ongoing nationally longitudinal study, and surveys began in 1989, with subsequent exams every 2 to 4 years, for a total of 9 rounds between 1989 and 2011. CHNS was designed to examine the effects of the health and nutrition in both urban and rural China, and to see how the economic, demographic, and social factors affected health and nutritional status of the Chinese population. A stratified multistage, random cluster sampling procedure was employed to draw the sample from 9 provinces (Heilongjiang, Liaoning, Shandong, Jiangsu, Henan, Hubei, Hunan, Guizhou, and Guangxi) and 3 autonomous regions (Beijing, Shanghai, and Chongqing), that vary substantially in terms of geography, economic level, public resources, and health indicators. Details about the CHNS have been described previously.^[19]^ Each participant has given a written informed consent and the study was approved by institutional review board from the University of North Carolina at Chapel Hill and the National Institute for Nutrition and Food Safety, China Center for Disease Control and Prevention. A total of 12,976 adult participants aged over 18 years old were obtained in the latest dataset of CHNS 2011.

### 2.2 Dependent and independent variables

The dependent variable in our analysis is utilization of preventive care in the last 4 weeks. The survey asked, “During the past 4 weeks, did you receive any preventive health service?” Those who received treatment were further asked, “What service did you receive?” Respondents were classified into 2 categories, subjects who did not use preventive care at all and subjects who used at least 1 preventive care service last 4 weeks.

Independent variables were selected on the basis of behavioral model of health service utilization established by Aday and Andersen.^[20,21]^ This model was frequently used to analyze the factors associated with access to healthcare and utilization of healthcare services.^[22,23]^ In this model, we classified age, gender, education, and marital status as predisposing factor; family income and health insurance status as enabling factor; and self-perceived illness in last 4 weeks and presence of physician-diagnosed chronic diseases (hypertension, diabetes, and others) as health status factor. Dependent and independent variables description are shown in Table 1.

**Table 1:**
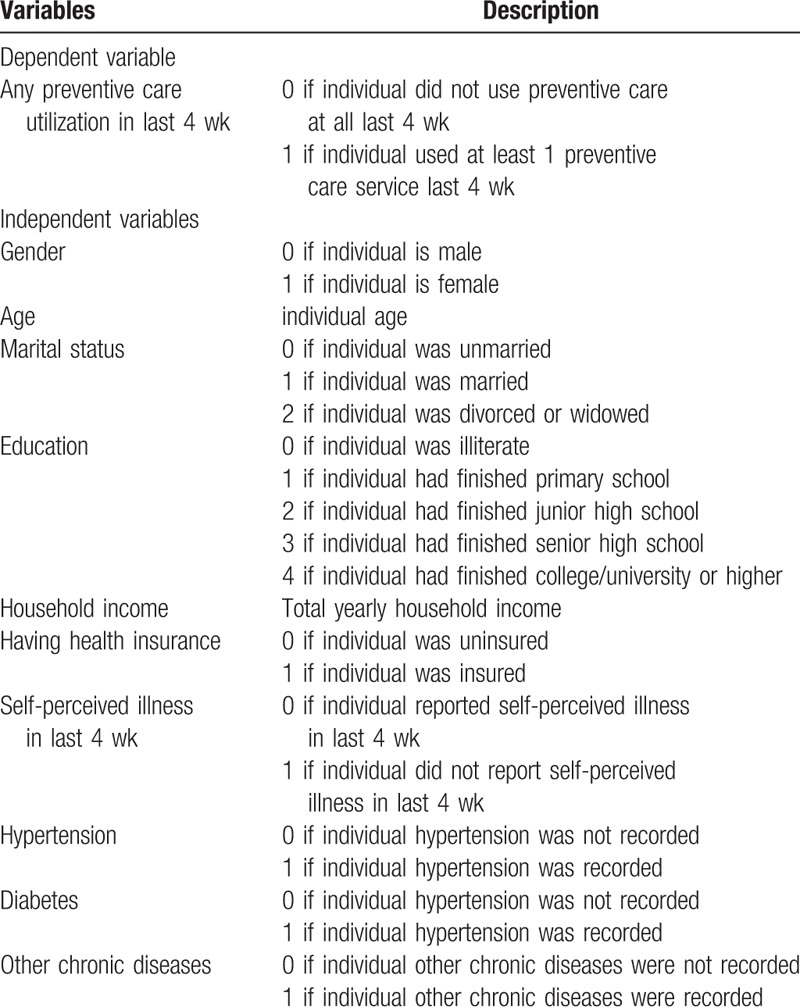
Variables description.

### 2.3 Statistical analysis

Firstly, we used Chi-square test to compare the utilization of preventive care services as well as other explanatory variables between urban and rural residents. Secondly, multilevel logistic regression model was used to study urban–rural disparity in utilization of preventive care services after controlling for the confounding variables; random intercepts were fitted for household level to adjust clustering of individuals within family. The equation we used for multilevel logistic regression model was^[24]^: 
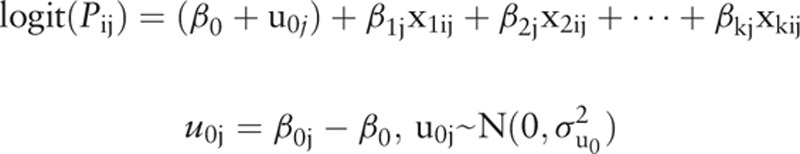


In the model, family was set to level 2, and individual was set to level 1. x_ij_ denoted the value of independent variable for individual i on family j, β_1j_,β_2j_,⋯β_kj_ represented the regression coefficients for independent variables. β_0_ was the overall mean of logit (P_ij_) (across all families). u_0j_ was the difference between family j's mean and the overall mean, and u_0j_ was also consider as random effect. β_0_+u_0j_ was the mean of logit(P_ij_) for family j.

Finally, we applied Blinder–Oaxaca decomposition method to divide urban–rural disparity in utilization of preventive care services into 2 parts.^[25,26]^ One was observed part that can be explained by differences in covariates including predisposing factor, enabling factor, and health status factor. Another was unobserved residual part that cannot be accounted for by observed differences in the covariates. Blinder–Oaxaca decomposition can be written as^[24,25]^: 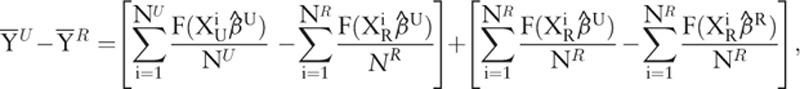


where  
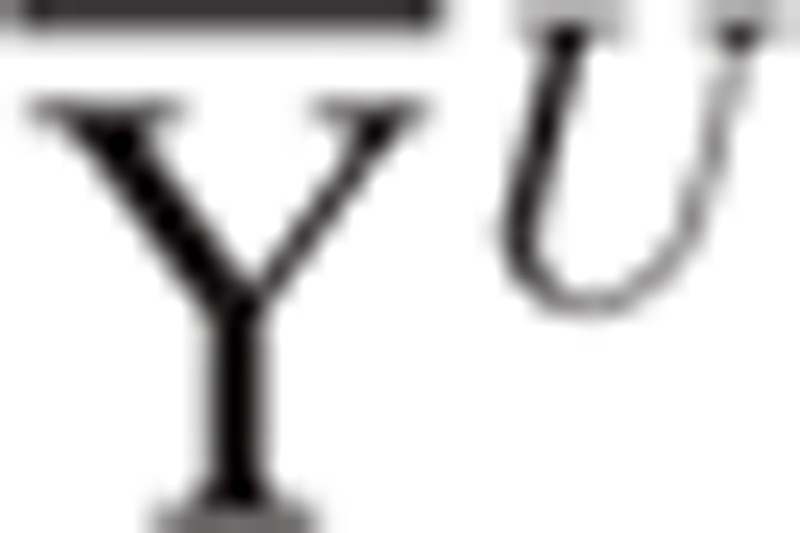
 and  
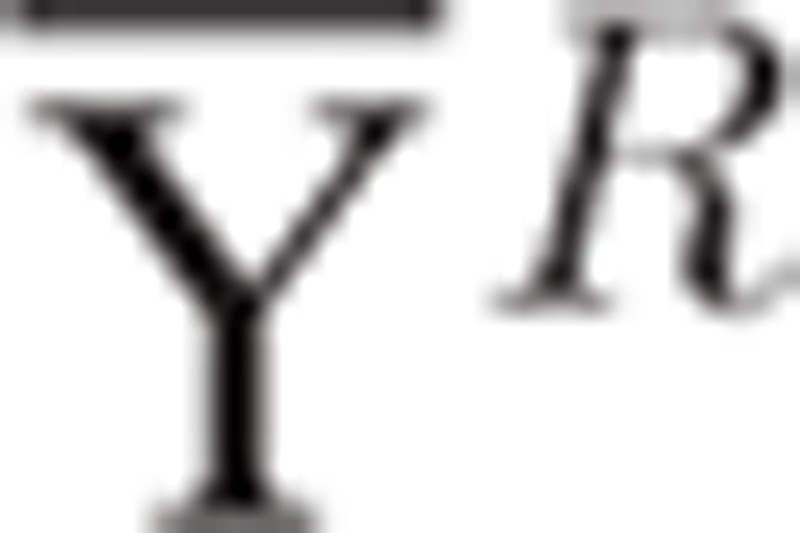
 was the average probability of preventive care utilization for urban and rural, N^U^ and N^R^ was the sample size for urban and rural.  
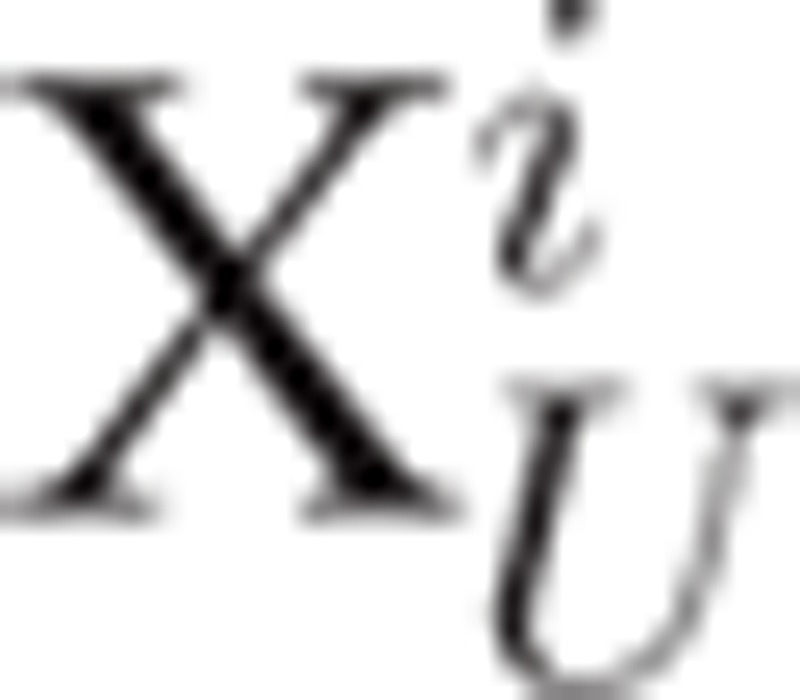
 and  
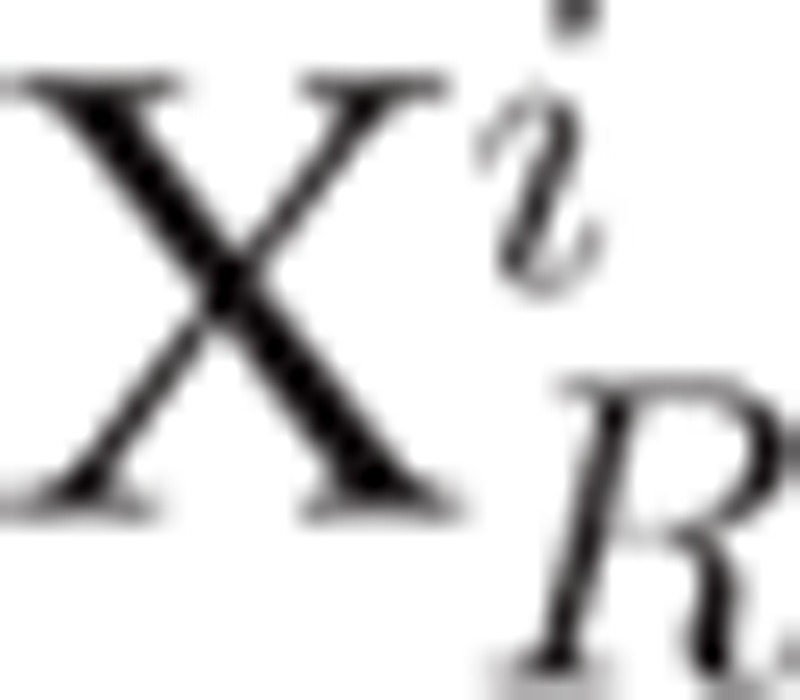
 was a row vector values of the independent variables for urban and rural,  
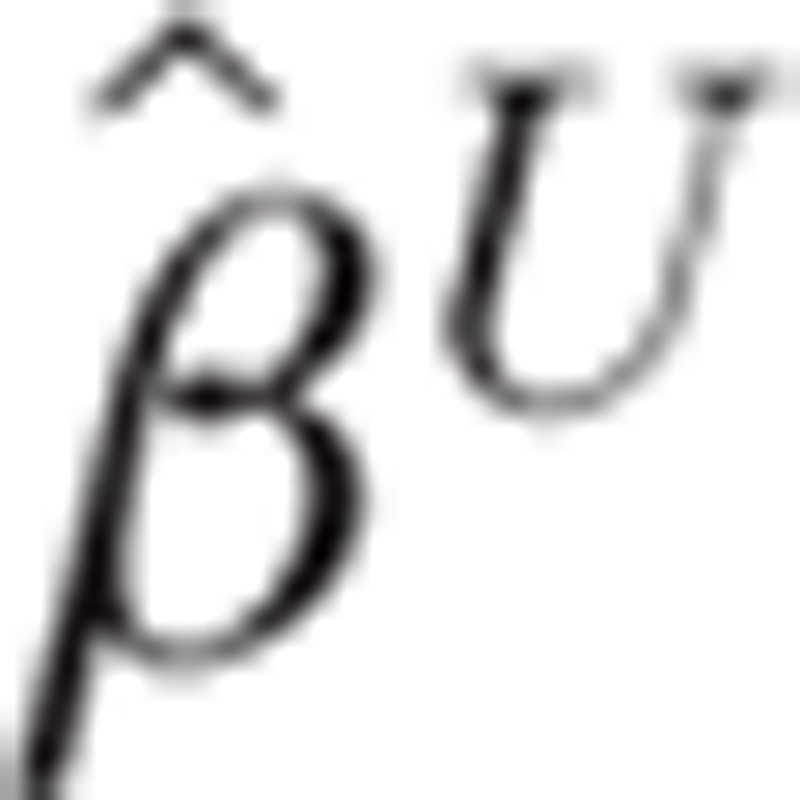
 and  
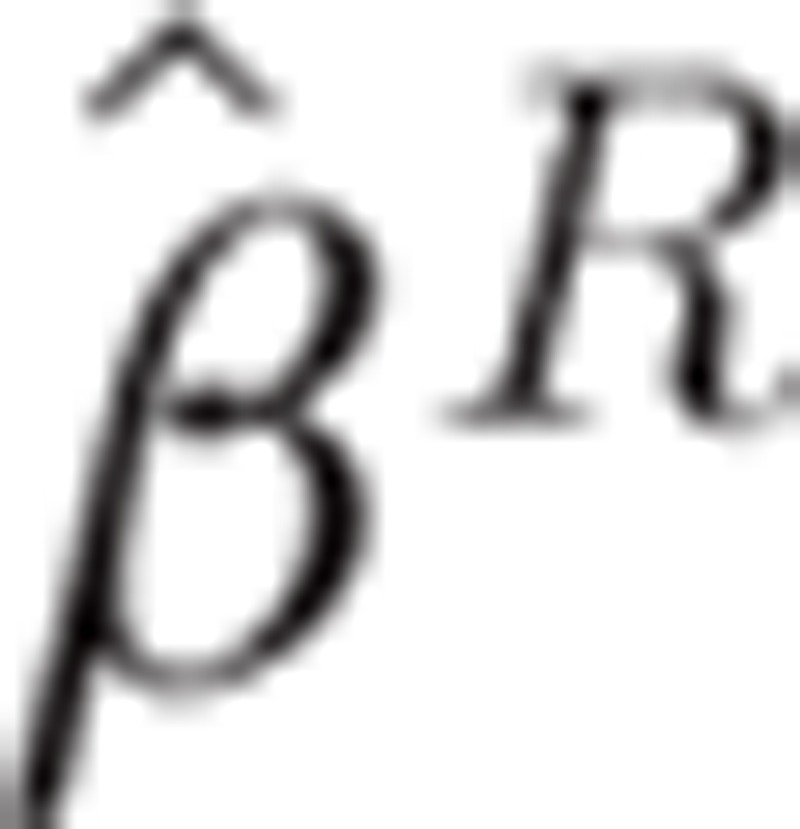
 was a vector of coefficient estimates for urban and rural. F was the cumulative distribution function from the logistic distribution. The first term in brackets represented the part of the urban–rural gap that was due to the group differences in distributions of independent variables, and the second term in brackets captured the portion of the urban–rural gap due to group differences in immeasurable or unobserved endowments. A *P*-value 0.05 was considered statistically significant. All data management and statistical analyses were performed with SPSS 21.0 (descriptive analyses), MLwinN 2.18 (multilevel model), and Stata 12.0 (Blinder–Oaxaca decomposition).

## 3 Results

### 3.1 Descriptive analysis

A total of 12,976 adult participants (6539 in urban and 6437 in rural) aged over 18 were investigated. Table 2 presents the descriptive statistics of variables used in this study for the urban and rural samples. The rate of utilization of preventive care services in last 4 weeks in urban residents was significantly higher than that in rural residents (9.3% vs 5.1%). General physical examination was the main type of preventive care service utilized by urban and rural residents. Among the residents who used preventive care service in last 4 weeks, more than half utilized general physical examination. Other types of utilized preventive care were examinations for specific conditions, such as tumor screening, blood pressure screening, vision or hearing examination, prenatal examination, gynecological examination, etc.

**Table 2:**
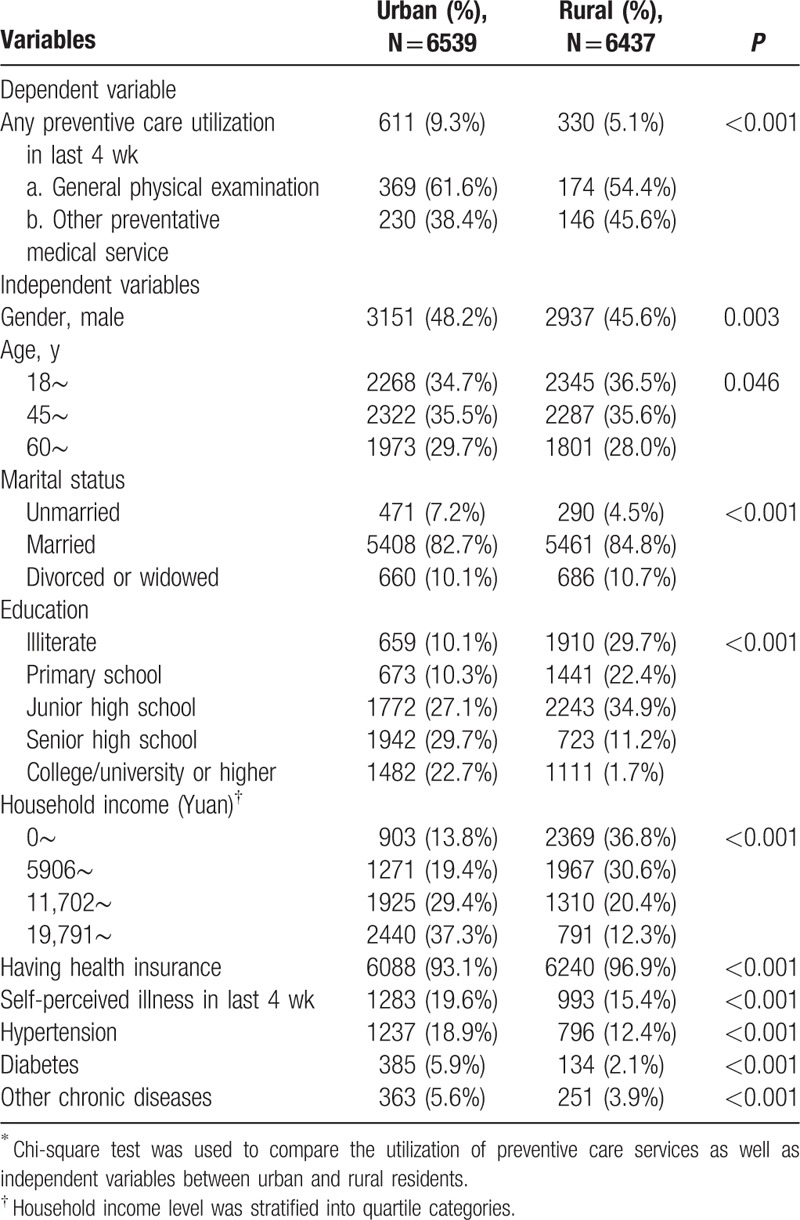
Summary statistics among rural and urban residents^∗^.

Compared with rural respondents, urban respondents were more likely to be male, older, unmarried, higher education, and household income, but less likely to have health insurance. A higher proportion of urban residents reported self-perceived illness in last 4 weeks (19.6% vs 15.4%). The prevalence of physician-diagnosed chronic diseases (hypertension, diabetes, and others) was higher among urban residents than among rural residents (Table 2).

### 3.2 Multivariate analysis

We presented 2 different multilevel logistic regressions for preventive care services (Table 3). The basic model (model 1) comprised only site variable (urban/rural). In full model (model 2), all independent variables were entered into the regression analysis along with the site variable. We reported odds ratios (OR) from each multilevel logistic regression.

**Table 3:**
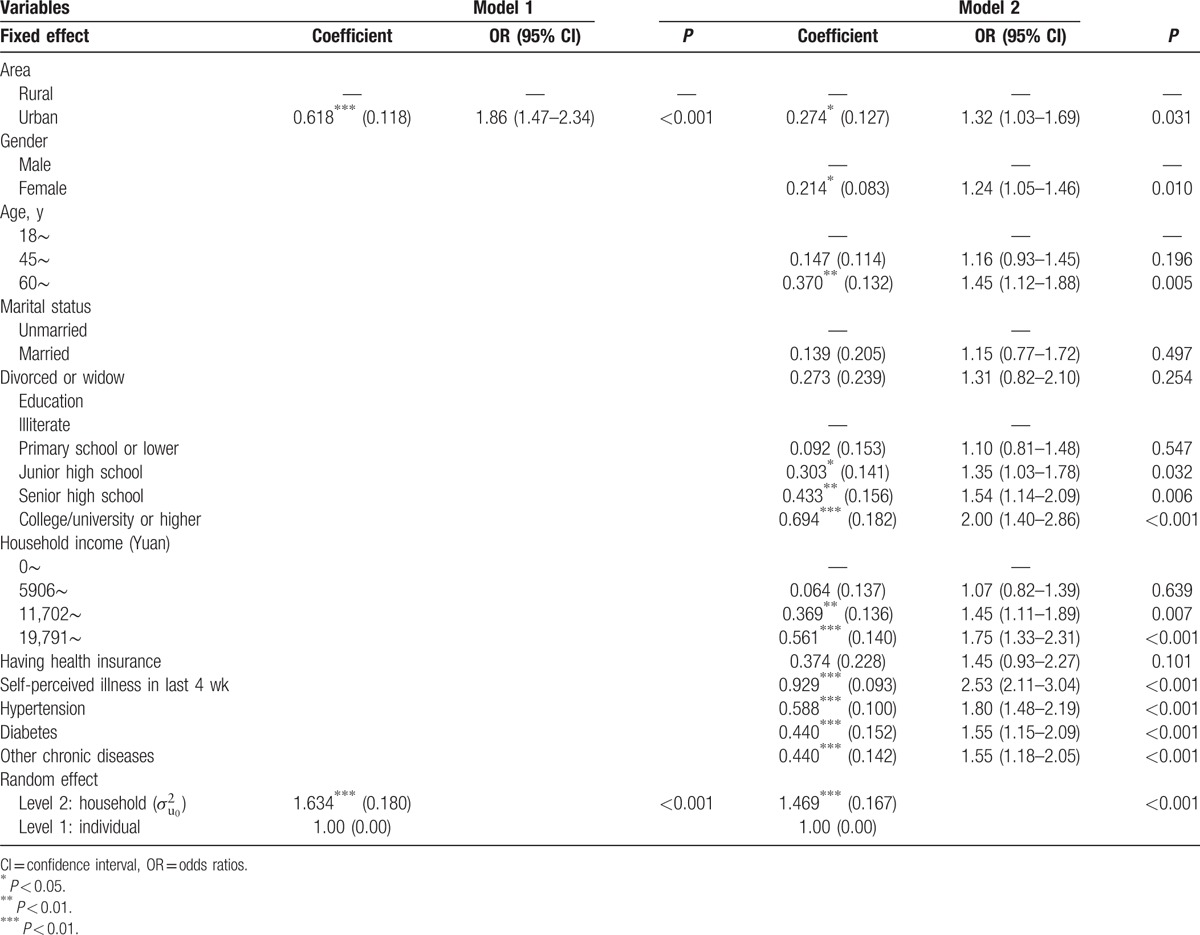
Multilevel logistic regression analyses for utilization of preventive care services.

In model 1 which included only site variable, urban residents had higher likelihood of utilizing preventive care services than rural residents (OR = 1.86; 95% CI: 1.47–2.34). After adjusting for all independent variables in model 2, the odds ratio for site decreased from 1.86 to 1.32, but it was still significant. This indicated that urban residents were still more likely to utilize preventive care services (OR = 1.32; 95% CI: 1.03–1.69) after controlling for the potential confounders. Additionally, gender, age, education level, household income, and health status were significantly associated with utilization of preventive care services. Female, the aged, residents with higher education level and household income, residents reporting self-perceived illness in last 4 weeks and physician-diagnosed chronic diseases (hypertension, diabetes, and others) had higher likelihood of utilizing preventive care services (Table 3).

### 3.3 Decomposition analyses

Table 4 provides the results using Blinder–Oaxaca decomposition technique to determine the relative importance of observed and unobserved components in accounting for urban–rural disparities. The results showed that both observed and unobserved components were significant, though the former were more important. Among observed determinants, household income exhibited the largest explanatory power, with a factor inequality weight of 27.1%, followed by education (21.5%), self-perceived illness in last 4 weeks (7.5%), hypertension (4.4%), diabetes (3.3%), other chronic diseases (0.8%), and health insurance (−1.0%). Gender, age, and marital status had sizeable effects, but they did not contribute to the explanation. Of the 3 explanatory factors, enabling factor was the most important component accounting for 26.1% of urban–rural disparities, followed by predisposing factor (22.7%) and health status factor (16.1%). All observed determinants contributed 64.9% of urban–rural disparities, and the remaining 35.1% was explained by unobserved component.

**Table 4:**
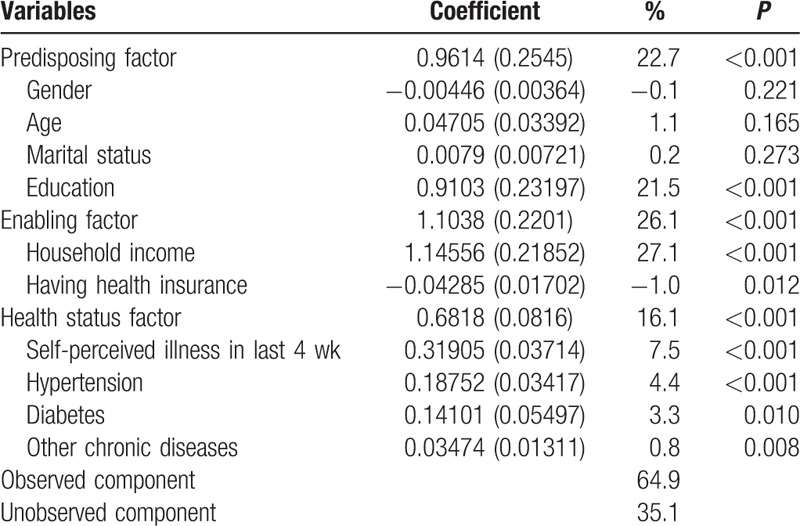
Blinder–Oaxaca decomposition results between urban and rural residents.

## 4 Discussion

The present study showed that rural residents were less likely to utilize preventive care services than urban residents (5.1% vs 9.3%). Previous study using CHNS data in 2009 showed that the rate of utilization of preventive care services were 3.9% and 4.8% respectively for rural and urban residents.^[16]^ Although utilization of preventive care services increased from 2009 to 2011 for both urban and rural residents, the gap in utilization of preventive care services between rural and urban children widened. To our knowledge, this is the first study to explore the contribution of each explanatory factor to urban–rural disparity in utilization of preventive care services.

After adjusting for explanatory variables, the odds ratio for site decreased from 1.86 to 1.32, which suggested that the explanatory variables we included in the model explained part of the urban–rural disparity in utilization of preventive care services. Of the 3 explanatory factors, enabling factor is the most important component contributing 26.1% of urban–rural disparity. The contribution of enabling factor is almost completely attributed to household income. Household income has been identified as an essential factor in the utilization of preventive care service.^[27,28]^ Preventive care services are generally consider to be inexpensive when compared with disease treatment, but in China, financial burden may still be a barrier for the utilization of preventive care due to decreased disposable income in the poor. Empirical evidence indicates that a large portion of disposable income is spent on basic living consumption including food and house in low-income family in China.^[29]^ It is very common that low-income people underutilize necessary medical care and forsake preventive care services because of their limited disposable income.^[30]^ This is alarming given the fact that urban–rural wealth gap is great in China, and rural residents are much poorer than urban residents on average, which contributed to urban–rural utilization inequality. Another enabling factor, health insurance, explained −1.0% of urban–rural disparity. This indicated that health insurance slightly narrowed the gap in utilization of preventive care between urban and rural. In our study, we found that rural residents were more likely to be insured than urban residents (96.9% vs 93.1%), which reflected the rapid increase in New Cooperative Medical Scheme (NCMS) coverage in rural.^[31]^ The NCMS is a mutual help and risk-pooling health protection scheme for rural residents. Although it is a voluntary insurance scheme, governments contribute the majority (more than 75%) of fund, and contributions from the insured are very limited. Its coverage focuses on catastrophic illness for inpatient and outpatient services. In 2011, the NCMS covered nearly every rural resident in China. However, despite the rapid expansion of insurance coverage in rural, the result of multilevel logistic regression model showed that there was no evidence that health insurance has promoted the utilization of preventive care services. This indicates that the current health insurance system in China is unable to play a significant role in reducing inequality in utilization of preventive care. Until now, insurance policy in China has focused on inpatient cost,^[32,33]^ with little concern for preventive health services. Health insurance does not cover most of preventive care services, such as general physical examination, tumor screening, vision or hearing examination, etc., either for NCMS or urban resident/employee health insurance. Our findings suggest that extending health insurance coverage may have limited success in promoting the utilization of preventive services, and providing more preventive care services covered by health insurance should be taken into consideration.

Predisposing factor played a vital role in urban–rural disparity of preventive services utilization, though it is slightly less important than enabling factor. Gender, age, and education were significantly associated with the utilization of preventive care services. Like previous studies,^[34–36]^ our study showed that female, the aged were more likely to utilize preventive services. There are several possible explanations for this. First, gender difference in utilization may be due to their attitudes concerning their own vulnerability between males and females.^[37]^ Stereotypes contribute to strongly held societal beliefs that male are stronger, tougher, and more robust than female, as well as beliefs that are consistent with men's own perceptions of themselves as being invulnerable.^[38]^ Also, the aged might utilize preventive care services more because the aged are in fact at a greater risk for disease and death than young people. Second, basic public health service system in China may promote higher utilization of preventive services in female and the aged. Some services are available to the general public, such as personal health records and health education. But most are selective, targeting a specific group of population including women (e.g., postnatal home visits and breast cancer screening) and elderly (e.g., general physical examination and diabetes screening).^[39]^ Other people who are not covered by the basic public health service packages would have to pay for such services, which may lead to decreased utilization of preventive services. It is worth noting that there were still statistically different proportions in gender and age between urban and rural, and urban residents were more likely to be male, older, but they were not meaningful in terms of the magnitude of difference (less than 3%). Therefore, gender and age hardly contributed to the explanation of urban–rural disparity in preventive services utilization. Another predisposing factor, education level, was essential for urban–rural disparity in preventive services utilization, which was next in importance to household income (contributed 27.1% and 21.5% descriptively). Economic explanation about the education effect on health service utilization is that education is related to income or occupational choice. This may explain only a part of the education effect. After income variable was controlled in our multilevel logistic regression model, education was still significant. Besides income, education is related to health beliefs including the values, attitudes, and knowledge that individuals possess regarding health and healthcare services which influence their perception of need of health services.^[18]^ Compared with urban residents, rural residents were less educated, which may result in decreased health belief about preventive health services,^[40,41]^ and consequently underutilizing preventive care services even if residents can afford them. Given the strong impact of health beliefs on prevention choices, we would recommend policies aimed at improving individual knowledge and awareness about health. In many countries, especially in those with free preventive care policy, policy intervention on educational and cultural grounds could be desirable in order to increase the utilization level of prevention.^[42]^

The contribution of health status factor was less than predisposing factor and enabling factor, but it was still significant, accounting for 16.1% of urban–rural disparity. The present study found that a higher proportion of urban residents reported self-perceived illness in last 4 weeks. The prevalence of hypertension, diabetes, and other chronic diseases were higher among urban residents than among rural residents. This does not mean health status of urban residents is worse than rural counterpart, and in fact urban residents are more sensitive to illness and discomfort, which lead to higher rate of self-perceived illness.^[43]^ In addition, urban residents have more accesses to health care that enhances accurate diagnosis of chronic diseases.^[44]^ Our study found that residents who reported self-perceived illness and physician-diagnosed chronic disease were more likely to utilize preventive services. Similar results also indicated that people are reluctant to seek health care unless impaired by health problems.^[45,46]^ Particularly, basic public health service packages in China also cover people with chronic diseases (e.g., management of hypertension, diabetes, and severe mental illness), which promoted chronic patients utilize public health service including preventive services. Because higher percentage of people reported health problems in urban area, this promoted urban residents to utilize preventive health services more frequently. This finding also suggests that residents who did not report health problems often neglect preventive care. In fact, all residents need preventive care services regardless of their health status. Furthermore, it does not mean residents are in good health status if they did not report health problems. So how to promote them to utilize preventive service more frequently should be paid more attention.

This study may be subject to some important limitations. First, preventive care services utilization was reported during the past 4 weeks, and there was no measure available in the CHNS data for a longer period, such as 1 year before the survey time. However, the period to measure the utilization of preventive care was the same for urban and rural residents, to some extent the study still can reflect the status of urban–rural disparity in utilization of preventive care. Second, self-reported illness and chronic diseases may not be a valid proxy of objective health status, but other health status indicators such as self-rated health and quality of life were not included in the questionnaire. Third, the variables we included in the model explained 64.9% of urban–rural disparities, and this highlighted the potential importance of other unobserved factors which explained 35.1% of the remaining difference. As the observed factors were characteristics of residents, the unobserved factors may be more involved in health service provider,^[44,45]^ such as price, quality (e.g., drug availability and vaccine availability), accessibility (e.g., distance), potential effect of region (e.g., health promotion campaign). And these factors were not included in the study design.

In conclusion, despite government interventions to increase access to and utilization of health services for rural residents in China, the present study shows that rural residents are still underutilizing preventive health services, when compared to urban counterparts. Household income, education level, and health status are important factors accounting for urban–rural disparity in utilization of preventive care services. A comprehensive approach to reduce the gap of utilization of preventive care services between urban and rural may focus on: reducing financial barriers for low-income residents who cannot afford preventive services; providing more preventive health services covered by health insurance; and increasing awareness of the importance and necessity of utilizing preventive health services.

## Acknowledgments

This research uses data from the China Health and Nutrition Survey (CHNS). We thank the National Institute of Nutrition and Food Safety, China Center for Disease Control and Prevention; the Carolina Population Center, University of North Carolina at Chapel Hill.
